# Supplementary material for: The Effects of Vehicle Type, Transport Duration and Pre-Transport Feeding on the Welfare of Sheep Transported in Low Temperatures
Source: Animals (Basel). 2021 Jun 2;11(6):1659. doi: 10.3390/ani11061659 (PMC8227339; doi:10.3390/ani11061659)
Supplement: Supplementary file 1 [file animals-11-01659-s001.zip › animals-1236584-supplementary.pdf]

animals

Supplementary Materials

Supplementary Tables

Supplementary Table S1. Mean ± standard error of mean (min, max) values of measured indoor and outdoor temperatures and blood parameters at departure and arrival in experiment 1.

Variable	Enclosed vehicle		Open vehicle	
	Departure	Arrival	Departure	Arrival
Indoor temperature, °C				
Head	+2.3±0.6 (1.1, 7.6)	+16.9±0.6 (12.8, 19.7)	−1.3±0.9 (−4.6, 3.9)	+12.4±0.6 (9.2, 15.3)
Ear	+0.3±0.7 (−2.9, 4.5)	+9.4±1.1 (5.0, 15.3)	−1.0±0.7 (−4.2, 3.3)	+1.8±0.3 (0.7, 3.3)
Abdomen	+35.1±0.2 (33.8, 35.9)	+36.0±0.3 (34.1, 37.2)	+34.6±0.6 (30.0, 36.9)	+36.2±0.5 (32.5, 38.0)
Foot/pastern	+31.9±0.9 (26.5, 34.4)	+31.6±1.3 (21.4, 36.3)	+30.1±1.3 (21.1, 34.6)	+31.2±1.4 (22.4, 35.1)
Outdoor minimum temperature, °C				
Ear	−13.8±1.06 (−18.9; −7.5)	−5.6±0.7 (−8.2; −1.7)	−17.5±30.9 (−21.1; −12.5)	−6.8±0.9 (−11.2; −1.3)
Foot/pastern	−16.5±1.03 (−21.7; −12.8)	−10.5±0.6 (−16.2;7,6)	−16.5±0.8 (−19.5; −12.2)	−10.3±0.4 (−12.4; −7.9)
Blood parameters				
ACTH, ng/L	+98.2±4.6 (75.6, 117.0)	+90.7±3.9 (71.6, 110.9)	+78.0±5.9 (53.3, 107.7)	+88.5±5.0 (55.2, 113.9)
ALT, U/L	+24.4±1.2 (19.3, 31.2)	+23.0±1.2 (19.1, 31.3)	+24.4±0.5 (21.4, 26.5)	+24.2±0.9 (18.8, 28.1)
HSP, ng/L	+1667.5±47.7 (1407.2, 1846.3)	+1386.2±109 (490.4, 1642.3)	+1711.5±59.7 (1519.6, 2141.5)	+1664.1±59.1 (1421.9, 2070.0)
Cortisol, µg/L	32.2±1.9 (27.6, 38.7)	+35.6±1.5 (28.0, 42.4)	+34.2±1.5 (25.0, 40.8)	+36.1±1.5 (31.0, 47.8)
NEFA, mmol/L	+204.7±8.3 (164.7, 240.2)	+197.2±5.17 (173.7, 226.6)	+177.2±5.9 (149.8, 201.2)	+206.9±8.8 (154.5, 255.5)
LDH, IU/L	+255.4±4.8 (235.7, 275.4)	+251.5±7.2 (225.1, 298.3)	+247.9±6.8 (221.4, 291.0)	+258.2±6.9 (232.2, 304.2)
CA, ng/L	+115.7±4.6 (95.6, 132.3)	+109.0±3.8 (86.5, 131.2)	+110.4±2.5 (100.8, 124.7)	+111.3±4.05 (92.2, 132.9)
CK, IU/L	+32.6±0.7 (27.1, 35.3)	+32.9±1.05 (28.7, 37.9)	+28.7±1.2 (24.3, 37.2)	+29.5±0.6 (25.1, 31.3)

Glucose, mmol/L	+4.18±0.14<br>(3.51, 5.29)	+4.17±0.24<br>(3.53, 6.24)	+4.17±0.15<br>(3.43, 5.21)	+5.04±0.5<br>(3.59, 7.94)

ACTH = adrenocorticotropic hormone; ALT = alanine aminotransferase; HSP = heat shock protein; NEFA= free fatty acid levels; LDH = lactic acid dehydrogenase; CA = catecholamine; CK = Creatine kinase.

Supplementary Table S2. Mean ± standard error mean (min, max) values of measured indoor and outdoor temperatures and blood parameters at departure and arrival in experiment 2.

Variable	Duration of transport 1 hour		Duration of transport 2 hours	
	Departure	Arrival	Departure	Arrival
Indoor temperature, °C				
Head	+13.7±0.4<br>(11.8, 15.8)	+24.0±1.2<br>(19.0, 28.8)	+14.4±0.6<br>(11.5, 18.0)	+23.4±1.1<br>(18.6, 28.9)
Ear	+4.0±0.8<br>(1.4, 9.6)	+10.2±1.3<br>(4.3, 17.9)	+9.6±1.1<br>(4.5, 15.6)	+8.0±1.2<br>(3.4, 15.9)
Abdomen	+36.2±0.3<br>(35.1, 37.7)	+37.3±0.26<br>(35.9, 38.5)	+35.7±0.2<br>(35.0, 36.9)	+36.5±0.4<br>(34.5, 37.9)
Foot/pastern	+32.4±0.7<br>(29.0, 35.4)	+32.7±0.6<br>(29.7, 35.1)	+33.5±0.43<br>(31.8, 36.3)	+34.6±0.6<br>(31.0, 36.4)
Outdoor minimum temperature, °C				
Ear	−5.7±0.7<br>(−9.3; −1.3)	−7.03±1.07<br>(−10.7; −1.8)	−3.5±0.6<br>(−6.7; 0.3)	−3.78±0.7<br>(−7.2; −0.8)
Foot/pastern	−6.09±1.2<br>(−11.9 ;1.9)	−11.1±0.9<br>(−17.3; −7.8)	−6.8±1.2<br>(−11.3; 1.9)	−7.56±0.7<br>(−9.7; −1.6)
Blood parameters				
ACTH, ng/L	+97.4±4.4<br>(82.6, 118.8)	+88.0±4.7<br>(67.0, 114.6)	+89.4±4.4<br>(69.9, 119.0)	+103.7±6.8<br>(60.8, 137.8)
ALT, U/L	+24.2±1.2<br>(19.4, 31.3)	+25.5±0.8<br>(22.1, 29.3)	+25.0±1.01<br>(21.0, 32.5)	+31.0±1.05<br>(26.5, 36.5)
HSP, ng/L	+1740.1±67.37<br>(1514.6, 2218.8)	+1584.5±54.6<br>(1360.1, 1930.6)	+1673.5±69.4<br>(1413.3, 2168.7)	1574.7±49.2<br>(1330.2, 1752.8)
Cortisol, μg/L	+37.8±2.4<br>(18.7, 45.0)	+39.3±1.05<br>(35.0, 45.0)	+40.6±1.1<br>(36.7, 45.4)	+44.4±1.09<br>(36.5, 48.2)
NEFA, mmol/L	+234.4±9.6<br>(188.2, 291.4)	+214.7±9.7<br>(177.7, 267.4)	+234.4±7.5<br>(194.8, 265.8)	+255.8±9.07<br>(210.44, 295.17)
LDH, IU/L	+255.9±8.7<br>(212.3, 306.5)	+248.0±8.9<br>(203.9, 288.4)	+301.6±14.7<br>(249.6, 378.4)	+329.4±14.8<br>(272.6, 409.0)
CA, ng/L	+119.1±3.8	+126.8±3.65	+141.5±5.14	+113.0±9.4

	(99.4, 136.6)	(110.6, 145.8)	(110.6, 164.4)	(79.2, 162.2)
CK, IU/L	+34.5±0.8	+35.4±1.03	+35.4±1.2	+40.4±1.3
	(29.8, 38.5)	(31.8, 40.2)	(29.8, 41.7)	(31.8, 46.8)
Glucose, mmol/L	+3.68±0.13	+3.65±0.3	+3.46±0.1	+4.26±0.6
	(3.08, 4.42)	(2.99, 5.99)	(2.94, 4.08)	(3.06, 9.27)

ACTH = adrenocorticotropic hormone; ALT = alanine aminotransferase; HSP = heat shock protein; NEFA= free fatty acid levels; LDH = lactic acid dehydrogenase; CA = catecholamine; CK = Creatine kinase

Supplementary Table S3. Mean ± standard error mean (min, max) values of measured indoor and outdoor temperatures and blood parameters at departure and arrival in experiment 3.

Variable	Yes		No	
Pre-transport feeding	Departure	Arrival	Departure	Arrival
Indoor temperature, °C				
Head	+11.6±0.5	+22.4±1.5	+13.7±0.7	+24.8±1.2
	(9.6, 13.4)	(15.8, 29.0)	(9.9, 17.6)	(18.7, 29.7)
Ear	+3.5±1.0	+7.6±0.9	+5.3±0.7	+10.8±2.2
	(0.2, 9.3)	(2.9, 11.8)	(1.7, 8.8)	(3.8, 26.6)
Abdomen	+35.1±0.2	+36.6±0.3	+36.1±0.4	+36.7±0.4
	(34.1, 36.2)	(34.7, 37.4)	(34.5, 37.9)	(34.3, 37.7)
Foot/pastern	+31.9±0.7	+31.7±1.1	+31.9±1.2	+33.9±0.6
	(27.2, 34.1)	(27.1, 35.8)	(26.4, 36.1)	(31.1, 37.6)
Outdoor minimum temperature, °C				
Ear	−13.4±0.7	−7.2±1.5	−12.1±1.5	−7.4±1.6
	(−18.6; −10.7)	(−14.7;0.4)	(−17.4; −3.8)	(−14.3;1.1)
Foot/pastern	−14.1±0.8	−10.1±0.7	−15.9±0.5	−9.04±0.7
	(−18.1; -−10.9)	(−13.5; −6.8)	(−12.4; −13.2)	(−12; −4.4)
Blood parameters				
ACTH, ng/L	+69.5±4.3	+65.4±3.2	+71.2±4.04	+61.9±3.4
	(55.2, 95.1)	(52.4, 80.0)	(53.5, 90.0)	(48.1, 80.4)
ALT, U/L	+21.5±2.0	+19.2±0.6	+22.6±1.6	+26.6±1.2
	(15.6, 33.9)	(16.8, 22.5)	(18.0, 34.4)	(21.9, 35.2)
HSP, ng/L	+2034.6±105	+2127.3±69	+2039.9±90.2	+2347.4±69.4
	(1687.3, 2738.5)	(1771.7, 2498.5)	(1478.9, 2454.4)	(1930.6, 2662.5)
Cortisol, μg/L	+23.1±3.7	+18.7±0.7	+22.3±1.4	+22.5±1.2
	(15.8, 45.1)	(15.6, 23.3)	(16.7, 30.0)	(17.6, 26.6)
NEFA, mmol/L	+174.5±9.0	+159.6±1.9	+184.5±9.8	+197.4±4.3
	(149.4, 243.7)	(150.1, 167.0)	(153.0, 257.0)	(181.2, 217.7)

LDH, IU/L	+274.4±10.9<br>(213.0, 315.8)	+252.9±14.5<br>(206.3, 342.4)	+268.5±16.1<br>(210.9, 347.0)	+298±12.5<br>(243.9, 364.6)
CA, ng/L	+123.8±8.9<br>(95.1, 173.4)	+116.5±4.6<br>(97.9, 139.7)	+126.0±5.8<br>(105.2, 162.5)	+133.7±4.3<br>(119.1, 167.3)
CK, IU/L	+41.5±3.2<br>(27.0, 63.7)	+39.8±1.9<br>(31.1, 53.1)	+41.5±1.7<br>(36.0, 50.6)	+45.2±1.9<br>(36.3, 58.3)
Glucose, mmol/L	+3.96±0.09<br>(3.62, 4.53)	+4.00±0.2<br>(3.40, 5.34)	+3.78±0.1<br>(3.23, 4.28)	+3.99±0.4<br>(3.26, 7.23)

ACTH = adrenocorticotropic hormone; ALT = alanine aminotransferase; HSP = heat shock protein; NEFA= free fatty acid levels; LDH = lactic acid dehydrogenase; CA = catecholamine; CK = Creatine kinase

Supplementary Figures

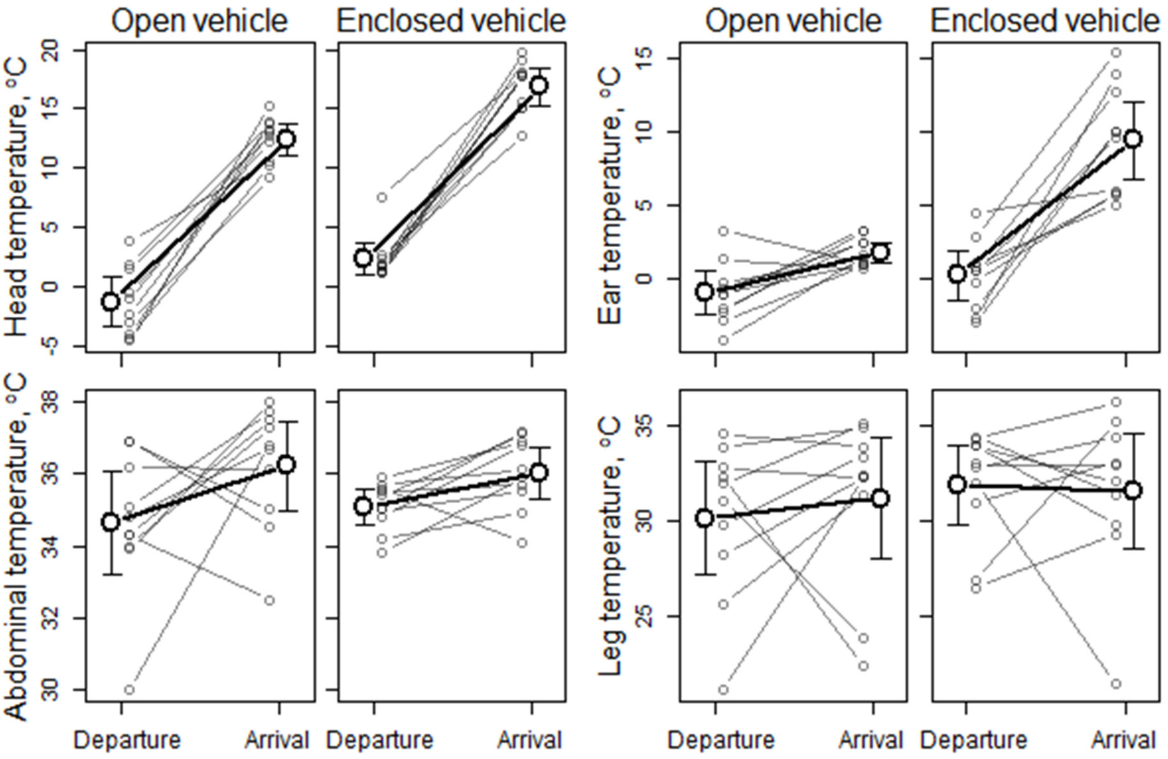

Supplementary Figure S1. Measured temperatures at departure and arrival in experiment 1 (animals exposed *vs* not exposed to the wind during transport). Larger circles with error bars denote mean values with 95% confidence interval, smaller circles joined with lines mark measurements made on single animals.

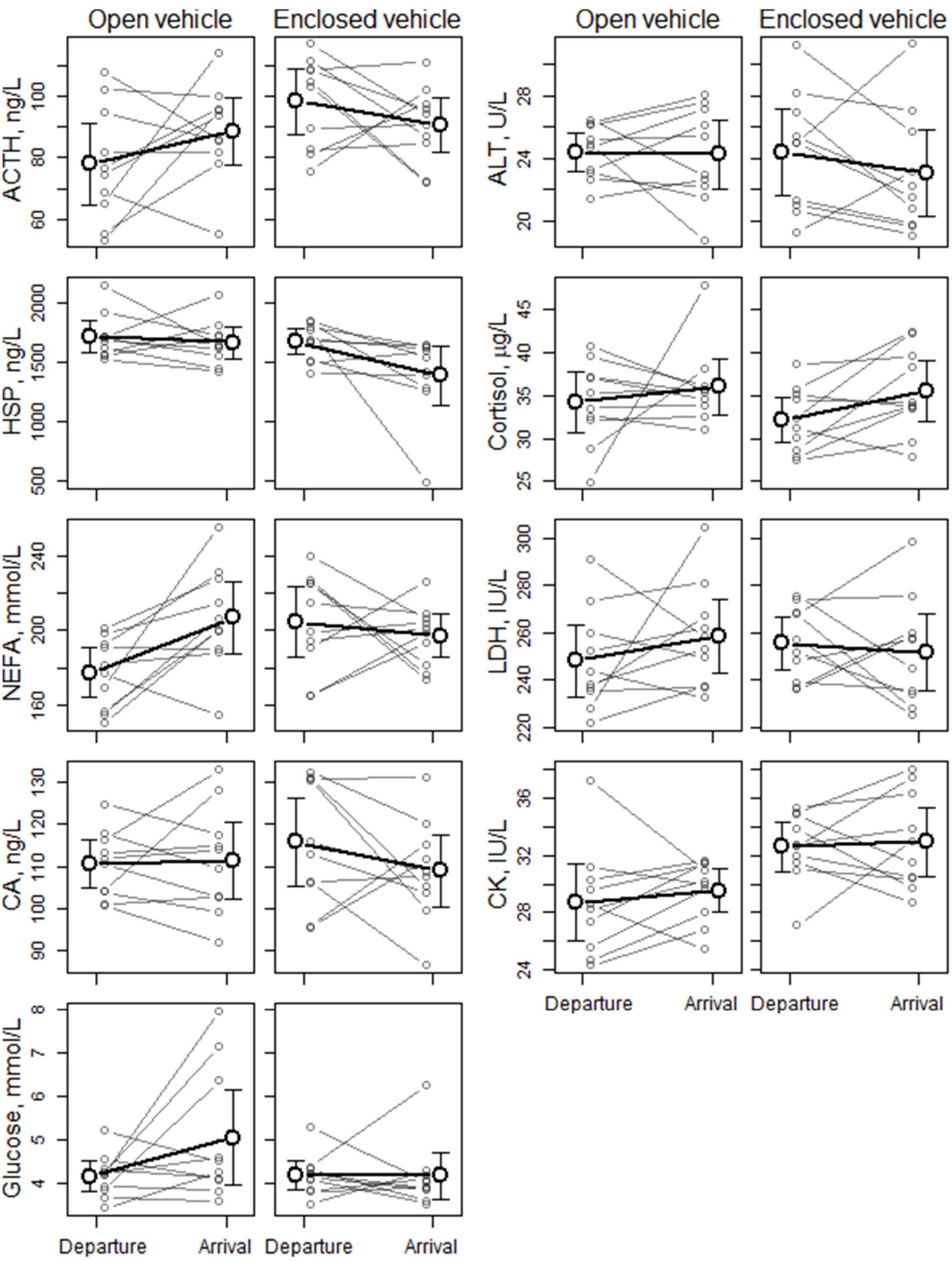

Supplementary Figure S2. Measured blood parameters at departure and arrival in experiment 1 (animals exposed *vs* not exposed to the wind during transport). Larger circles with error bars denote mean values with 95% confidence interval, smaller circles joined with lines mark measurements made on single animals.

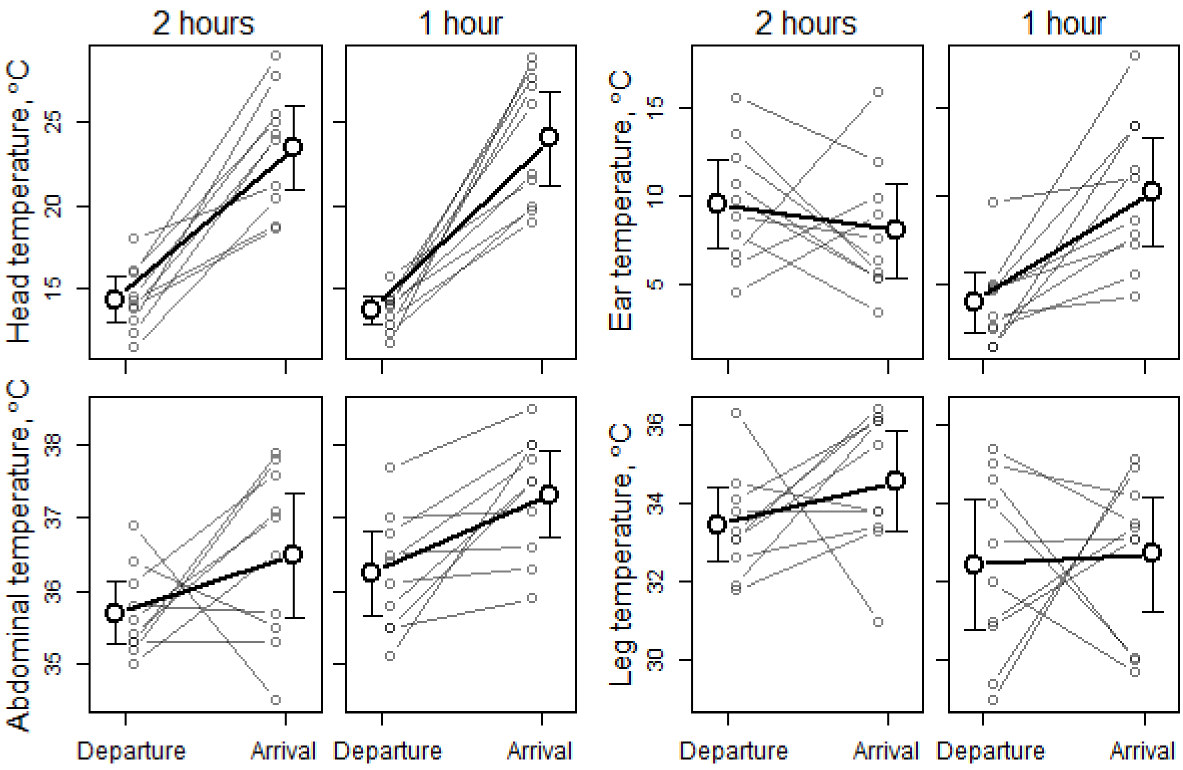

Supplementary Figure S3. Measured temperatures at departure and arrival in experiment 2 (duration of transport 2 hours *vs* 1 hour). Larger circles with error bars denote mean values with 95% confidence interval, smaller circles joined with lines mark measurements made on single animals.

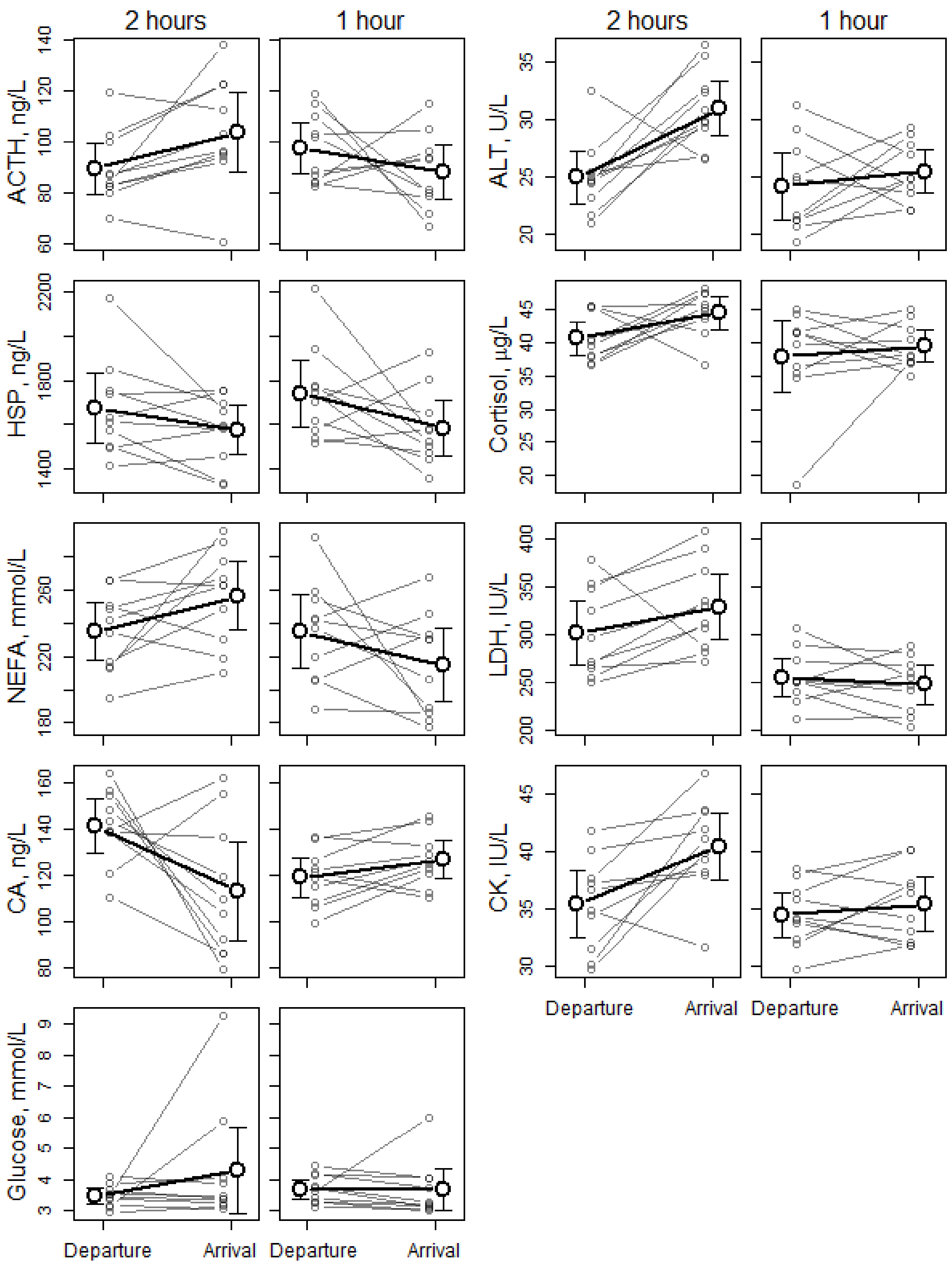

Supplementary Figure S4. Measured blood parameters at departure and arrival in experiment 2 (duration of transport 2 hours *vs* 1 hour). Larger circles with error bars denote mean values with 95% confidence interval, smaller circles joined with lines mark measurements made on single animals.

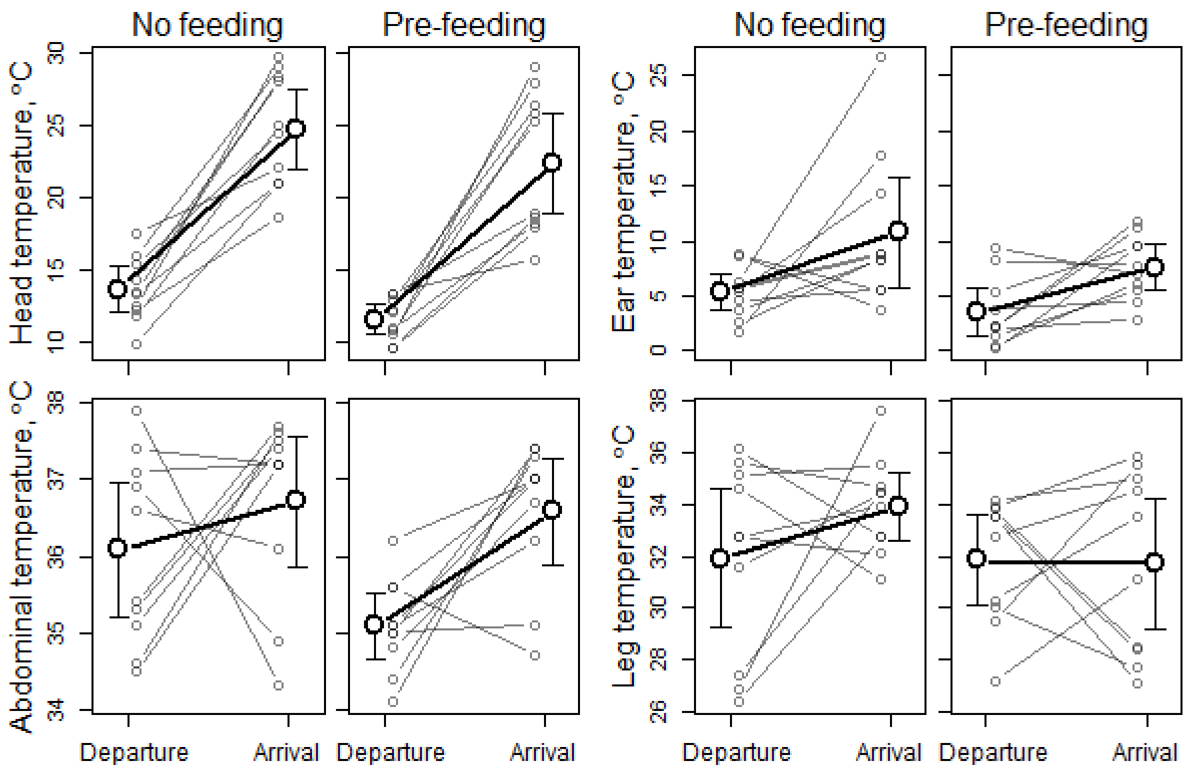

Supplementary Figure S5. Measured temperatures at departure and arrival in experiment 3 (without *vs* with pre-feeding). Larger circles with error bars denote mean values with 95% confidence interval, smaller circles joined with lines mark measurements made on single animals.

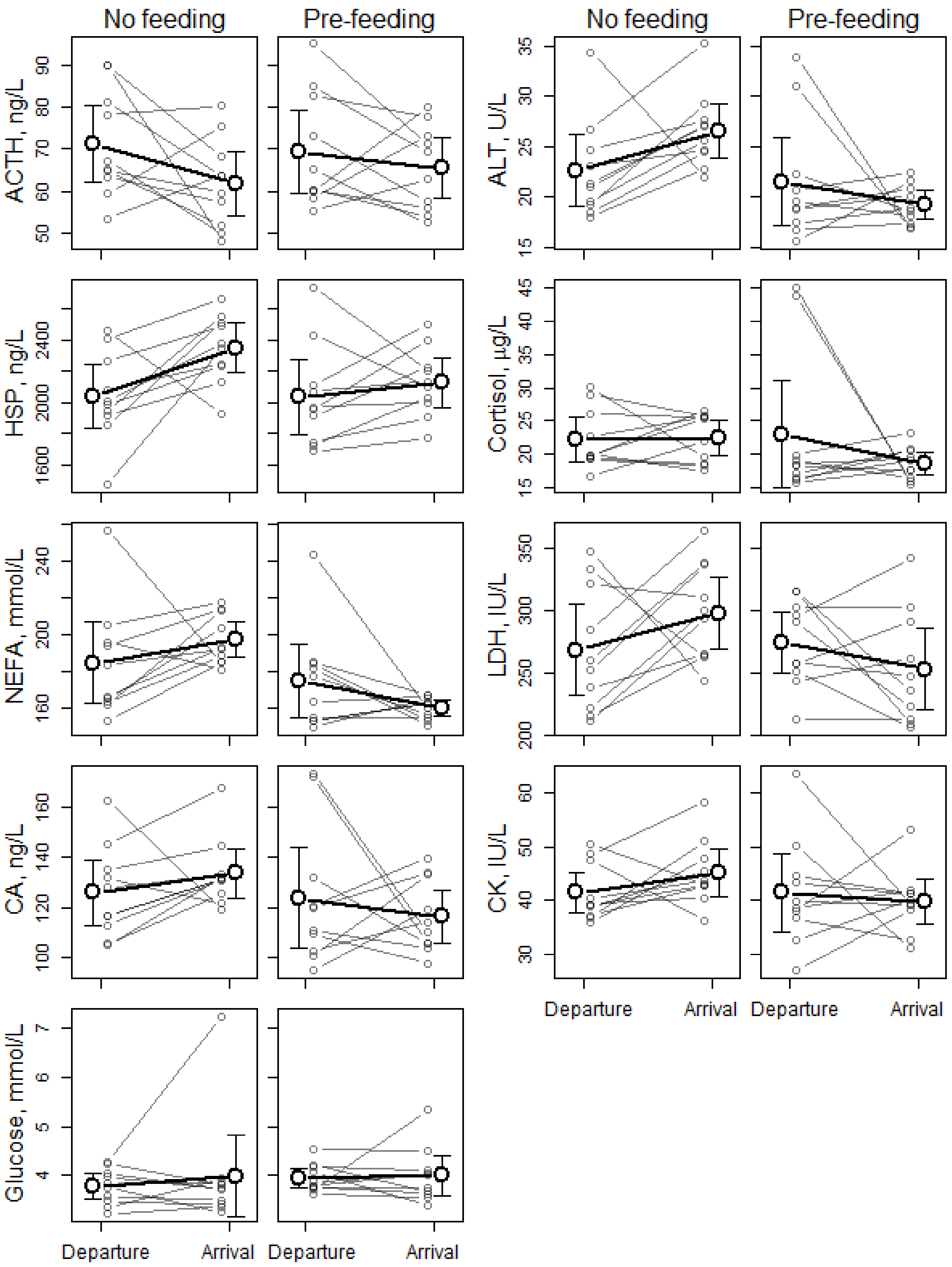

Supplementary Figure S6. Measured blood parameters at departure and arrival in experiment 3 (without *vs* with pre-feeding). Larger circles with error bars denote mean values with 95% confidence interval, smaller circles joined with lines mark measurements made on single animals.

Author Contributions: conceptualization, methodology, resources, BS, XJ and CJCP, supervision DA, collection of data FC, investigation and data curation, FC and TK, writing—original draft preparation, FC, DA and CJCP; writing—review and editing FC, CJCP and DA.

Funding: This research project was part of the International Animal Welfare Standards Project, which was funded by Open Philanthropy Project and administered by the Centre for Animal Welfare and Ethics, School of Veterinary Science, University of Queensland, Australia.

Institutional Review Board Statement: Sheep were cared for in accordance with the guidelines for animal experiments of Inner Mongolia Agricultural University. The experimental protocol (No: NND20212037) was approved by the Institutional Ethics Committee of Inner Mongolia Agricultural University.

Data Availability Statement: The raw data has not been published or stored elsewhere but is available on request from F.C.

Acknowledgments: We are grateful to Wenliang Guo, Lulu Shi, Shuo Yang, to help for the collection data, all student under the supervision of Dr Jin Xiao, Lecturer in College of Animal Science, Inner Mongolia Agricultural University.

Conflicts of Interest The authors declare no conflict of interest**.**

References

1. Broom, D.M. The welfare of livestock during road transport. In: M. Appleby, V. Cussen, L. Garcés, L. Lambert and J. Turner (Editors) Long distance transport and the welfare of farm animals. Chapter 7 pp 157−181. *Wallingford: CABI.* **2008**.
2. Cussen, M.C.; Garcés, V.A.; Lambert, L.; Turner, L.A.; Freer, J M; Dove, H and Nolan, JV. Nutrient requirements of domesticated ruminants. Melbourne: *CSIRO Publishing*; **2007**.
3. Messori, S.; Pedernera-Romano, C.; Rodriguez, P.; Barnard, S.; Giansante, D.; Magnani, D.; Dalmau, A.; Velarde, A.; Dalla Villa, P. Effects of different rest-stop durations at control posts during a long journey on the welfare of sheep. *Vet. Ital.* **2017**,53, 121–129.
4. Fisher, A.D.; Niemeyer, D.O.; Lea, J.M.; Lee, C.; Paull, D.R.; Reed, M.T.; Ferguson, D.M. The effects of 12, 30, or 48 hours of road transport on the physiological and behavioural responses of sheep. *J. Anim. Sci.* **2010**,88,2144–2152.
5. Tarrant, P.V.; Kenny, F.J.; Harrington, D.; Murphy, M. Long distance transportation of steers to slaughter: effect of stocking density on physiology, behaviour and carcass quality. *Livest. Prod. Sci.* **1992**, 30, 223–238.
6. Cockram, M.S.; Baxter, E.M.; Smith, L.A.; Bell, S.; Howard, C.M.; Prescott, R.J.; Mitchell, M.A. Effect of driver behaviour, driving events and road type on the stability and resting behaviour of sheep in transit. *Anim. Sci.* **2004**, 79, 165–176.
7. Ruiz-de-la-Torre, J.L.; Velarde, A.; Manteca, X.; Diestre, A.; Gispert, M.; Hall, S.J.G.; Broom, D.M. Effects of vehicle movements during transport on the stress responses and meat quality of sheep. *Vet. Rec.* **2001**; 148:227–229.
8. Collins, T.; Stockman, C.A.; Barnes, A.L.; Miller, D.W.; Wickham, S.L.; Fleming, P.A. Qualitative behavioural assessment as a method to identify potential stressors during commercial sheep transport. *Animals (Basel).* **2018** Nov 15;8(11):209.
9. Broom, D. M.; Goode, J. A.; Hall, S. J. G.; Lloyd, D. M. and Parrott R. F. Hormonal and physiological effects of a 15h road journey in sheep: comparison with the response to loading, handling and penning in the absence of transport. *Br. Vet. J.* **1996;** 152: 593-604.
10. Parrott, R.F.; Hall, S.J.G.; Lloyd, D.M. Heart rate and stress hormone responses of sheep to road transport following two different loading procedures. *Anim. Welf.*; **1998**, 7, 257–267.
11. Fisher, A.D.; Stewart, M.; Duganzich, D.M.; Tacon, J.; Matthews, L.R. The effects of stationary periods and external temperature and humidity on thermal stress conditions within sheep transport vehicles, *N. Z. Vet. J.*, **2005;** 53:1, 6-9.
12. Ames, D. R.; Insley, L. W. Wind-chill effect for cattle and sheep, *J. Anim. Sci,* Volume 40, Issue 1, January **1975**, Pages 161–165.
13. Wojtas, K.; Cwynar, P.; Kolacz, R. Effect of thermal stress on physiological and blood parameters in merino sheep. *Bull. Vet. Inst. Pulawy.* **2014**. 58.
14. Liang, X.; Jin, J.; Bi, X.; Kamruzzaman, M.; Kudo, T.; Sano, H. Effects of Chinese herbal medicine and cold exposure on plasma glucose, leucine and energy metabolism in sheep. *J. Anim. Physiol. Anim. Nutr.*; **2018**.102: e534–e541.
15. Freer, M.; Dove, H.; and Nolan, J. V. Nutrient Requirements of Domesticated Ruminants. *CSIRO Publishing: Melbourne, Vic.* ***2007.***
16. Cottle, D.J.; Pacheco, D. Prediction of fleece insulation after shearing and its impact on maintenance energy requirements of Romney sheep. *Small Ruminant Res.* **2017**; 157:14-22.
17. Zhang, X.Q.; Kemp, D.; Ma, Y.B.; Jiang, C. Effect of warm-shed feeding on liveweight loss and lambing performance of ewes during winter–spring period. *Acta Pratac. Sin* **2017**; 26:203-9.
18. Cockram, M.S.; Kent J.E.; Goddard, P.J.; Waran N.K.; McGilp, I.M.; Jackson, R.E.; Muwanga, G.M. and Prytherch, S. Effect of space allowance during transport on the behavioural and physiological responses of lambs during and after transport. *Anim. Sci.* **1996**. 62, 461–477.

19. Hall, S.J.G.; Bradshaw, R.H. Welfare aspects of the transport by road of sheep and pigs. *J. Appl. Anim. Welf. Sci*. **1998**, 1, 235–254.
20. Chambers, P.G.; Grandin, T.; Heinz, G.; Srisuvan, T. Guidelines for Humane Handling, Transport and Slaughter of Livestock,book chapter. FAO Regional Office for Asia and the Pacific. *Publication (FAO Regional Office for Asia and the Pacific)* **2001** FAO. Regional Office for Asia and the Pacific.
21. Rioja-Lang, F.C.; Brown, J.A.; Brockhoff, E.J.; Faucitano, L. A Review of Swine Transportation Research on Priority Welfare Issues: A Canadian Perspective. *Front. Vet. Sci.* **2019**-February-22.
22. Guàrdia, M.D.; Gispert, M.; Diestre, A. Mortality rates during transport and lairage in pigs for slaughter. *Meat Focus Inter.* **1996** 10: 362.
23. Peterson, E.; Remmenga, M.; Hagerman, A.D.; Akkina, J.E. Use of temperature, humidity, and slaughter condemnation data to predict increases in transport losses in three classes of swine and resulting foregone revenue. *Front Vet. Sci.* **2017** 4:67.
24. Gonyou, H.W.; Brown, J. Reducing stress and improving recovery from handling during loading and transport of market pigs. *Final report submitted to Alberta Livestock and Meat Agency,* Edmonton, AB **2012** p. 40.
25. Guàrdia, M.D.; Estany, J.; Balasch, S.; Oliver, M.A.; Gispert, M.; Diestre A. Risk assessment of PSE condition due to preslaughter conditions and RYR1 gene in pigs. *Meat Sci*. **2004** 67:471–8.
26. Knowles, T. G.; Brown, S.N.; Warriss, P. D.; Phillips, A. J.; Dolan, S. K.; Hunt, P.; Ford, J. E.; Edwards, J. E.; Watkins, P. E. Effects on sheep of transport by road for up to 24 hours. *Vet. Rec*. **1995** 136:431–438.
27. Knowles, T. G., P. D. Warriss, S. N. Brown, and S. C. Kestin. Long distance transport of export lambs. *Vet. Rec*. **1994** 134:107–110
28. Time and date **2020**. Available from: https://www.timeanddate.com/weather/china/hohhot/historic?month=1&year=2020 (accessed on 1 November 2020).
29. Taylor, R.E.: Adaptation to the environment. In: *Scientific Farm Animal Productio*n, Macmillan Publishing Company, New York, NY, **1992**, pp. 326–332.
30. Hales, J.R.S.; Bennett, J.W., Fawcett, A.A. Effects of acute cold exposure on the distribution of cardiac output in the sheep. *Pflugers Arch.* **1976** Nov 5; 366(2-3):153-7.
31. Webster, A.; Hicks, A.; Hays, F. Cold climate and cold temperature induced changes in the heat production and thermal insulation of sheep. *Can. J. Physiol. Pharmacol.* **1969**. 47. 553-62.
32. Ekesbo, I. Farm Animal behaviour: characteristic for assessment of health and welfare; *CAB International:* Oxfordshire, UK; Cambridge University Press: Cambridge, UK, **2011**; p. 237.
33. Dabiri, N.; Holmes, C.W.; McCutcheon, S.N.; Parker, W.J.; Morris, S.T. Resistance to cold stress in sheep shorn by covercomb or standard comb. *Anim. Sci.* **1995**, 60, 451–456.
34. Piirsalu, P.; Kaart, T.; Nutt, I.; Marcone, G.; Arney, D. The effect of climate parameters on sheep preferences for outdoors or indoors at low ambient temperatures. *Animals* **2020** 10, no. 6: 1029.
35. OMAFRA. Ministry of agriculture, food and rural affairs, Ontario Canada. **2016** Available from http://www.omafra.gov.on.ca/english/livestock/sheep/facts/02-013.htm (accessed on 1 November 2020).
36. Department of Primary Industries and Regional Development's Agriculture and Food. **2018** Available from: https://www.agric.wa.gov.au/animal-welfare/hypothermia-sheep (accessed on 1 November 2020).
37. Canadian Agri-Food Research Council. Recommended Code of Practice for the Care and Handling of Farm Animals - Transportation, **2001**. Available from: http://nfacc.ca/pdf/english/Transportation2001.pdf (accessed on 1 November 2020).
38. Obst, J.M. and Ellis, J.V. Weather, ewe behaviour and lamb mortality. *Agric. Rec*., **1977** 20: 44-49.
39. Lynch, J.J. and Alexander, G., The effect of gramineous wind breaks on behaviour and lamb mortality among shorn and unshorn Merino sheep during lambing. *Appl. Anim. Ethol*. **1976**, 2: 305-325.
40. Lynch, J.J. and Alexander, G., Sheltering behaviour of lambing Merino sheep in relation to grass hedges and artificial wind breaks. *Aust. J. Agric. Res*. **1977**, 28: 691-701.
41. Younis, F. Expression pattern of heat shock protein genes in sheep. *Mansoura Vet. Med. J* **2020**. 21. 1-5.
42. Sejian, V.; Maurya, V.P.; Sharma, K.C.; Naqvi, S. Concept of multiple stresses and its significance on livestock productivity. Environmental Stress and Amelioration in Livestock Production: *Springer*; **2012**. p. 129-50.
43. Agnew, L.L.; Colditz, I.G. Development of a method of measuring cellular stress in cattle and sheep. *Vet. Immunol. Immunopathol.* **2008** Jun 15;123(3-4):197-204.
44. Kapila, R. Effect of low temperature on metabolic enzymes and HSP-70 expression of cold-water fish Bariliusbendelisis. *Asian Fish. Sci.* **2009**. 22. 125-136
45. Eng, J. W.; Reed, C. B.; Kokolus, K. M.; Repasky, E. A. Housing temperature influences the pattern of heat shock protein induction in mice following mild whole-body hyperthermia. *International Journal of Hyperthermia: the Official Journal of European Society for Hyperthermic Oncology, North American Hyperthermia Group*, **2014**. *30*(8), 540–546.
46. Parrott, R.F.; Misson, B.H. and de la Riva, C.F. Differential stressor effects on the concentrations of cortisol, prolactin and catecholamines in the blood of sheep. *Vet. Sci. Res.* **1994** 56, 234–239.
47. Fordham, D. P.; Lincoln, G. A.; Ssewannyna, E.; Rodway, R. G. Plasma β-endorphin and cortisol concentrations in lambs after handling, transport and slaughter. *Anim. Prod.* **1989**. 49, 10,3.

48. Androine, I.; Parvu, M.; Androine, V. The effects of transport stress on sheep welfare. Lucrari Stiintifice - Zootehnie si Biotehnologii, Universitatea de Stiinte Agricole si Medicina Veterinara a Banatului Timisoara, **2008** 41(1):729-734.
49. Zhong, R.Z.; Liu, H.W.; Zhou, D.W.; Sun, H.X.; Zhao, C.S. The effects of road transportation on physiological responses and meat quality in sheep differing in age. *J. Anim. Sci.*, **2011** 89, 3742-3751.
50. Braun, J.P.; Trumel, C.; Bézille, P. Clinical biochemistry in sheep: a selected review. *Small Rumin. Res.,* **2010**. 92, 10-18.
51. Earley, B.; Murray, M. The effect of road and sea transport on inflammatory, adrenocortical, metabolic and behavioural responses of weanling heifers. *Vet. Res.*, **2010** 6, 36-48.
52. Miranda-De la Lama, G.; Monge, P.; Villarroel, M.; Olleta, J.; GarcãA-Belenguer, S.; Marã, A.G. Effects of road type during transport on lamb welfare and meat quality in dry hot climates. *Trop. Anim. Health Prod.*, **2011** 43, 915-922.
53. Beatty, D.T.; Blache, D.; Wemelsfelder, F.; Fleming, P.A. Flooring and driving conditions during road transport influence the behavioural expression of cattle. *Appl. Anim. Behav. Sci.* **2013**, 143, 18–30.].
54. Walter, G.; Smith, G.; Walker, R. Chapter 29 Interpretation of clinical pathology results in non-clinical toxicology testing. *Haschek and Rousseaux's Handbook of Toxicologic Pathology Book, Third Edition* **2013**. Academic Press. Cambridge, Massachusetts, Stati Uniti.
55. Hrkovic-Porobija, A.; Hodzić, A.; Hadzimusic, N. Functional liver stress in dairy sheep. *I.J.S.R. (The)* **2017**. 23. 194.
56. Ašimović, Z. Izabrani parametri metabolizma kaoindikatori specifičnosti dubskog soja pramenke. PhD thesis, University of Sarajevo, Sarajevo, Bosnia and Herzegovina. **2005.**
57. de Freitas, M.C.; Gerosa-Neto, J.; Zanchi, N.E.; Santos Lira, F.; Rossi, F.E. Role of metabolic stress for enhancing muscle adaptations: Practical applications. *World J. Methodol.* vol. 7,2 46-54. 26 Jun. **2017**.
58. Coffey, V.G.; Shield, A.; Canny, B.J.; Carey, K.A.; Cameron-Smith, D.; Hawley, J.A. Interaction of contractile activity and training history on mRNA abundance in skeletal muscle from trained athletes. *Am. J. Physiol. Endocrinol. Metab.* **2006** ;290: E849–E855.
59. Bórnez, R.; Linares, M.B.;Vergara, H.. Haematological, hormonal and biochemical blood parameters in lamb: effect of age and blood sampling time. *Livest. Sci.*, **2009** 121, 200-206.
60. Jones, A. R. & Prince S. E. *In the Biology of Deer*, pp. 211-216, ed. R. D. Brown. Berlin **1992**.: Springer-Verlag.
61. Boccardo, A.; Belloli, A.; Locatelli, V.; Morandi, N.; Baggiani, L.; Cavallone, E.; Palma, A.; Pravettoni, D. Effect of lairage after transport in lambs slaughtered with islamic ritual. *Large Anim. Rev.* **2014**. 20. 71-79.
62. Proctor, H.S.; Carder, G. Can changes in nasal temperature be used as an indicator of emotional state in cows? *Appl. Anim. Behav. Sci.* **2016** 184, 1-6.
63. Alsaaod, M.; Syring, C.; Dietrich, J.; Moherr, M.G.; Gujan, T.; Steiner, A. A field trial of infrared thermography as a non-invasive diagnostic tool for early detection of digital dermatitis in dairy cows. *Vet. J.* **2014** 199, 281-285.
64. Gómez, Y.; Bieler, R.; Hankele, A.K.; Zähner, M.; Savary, P.; Hillmann. E. Evaluation of visible eye white and maximum eye temperature as non-invasive indicators of stress in dairy cows. *Appl. Anim. Behav. Sci.* **2018** 198, 1-8
65. Schaefer, A.; Cook, N.; Bench, C.; Chabot, J.; Colyn, J.; Liu, T.; Okine, E.; Stewart, M.; Webster, J. The non-invasive and automated detection of bovine respiratory disease onset in receiver calves using infrared thermography. *Res. Vet. Sci.* **2012** 93, 928-935
66. Stokes, J.; Leach, K.; Main, D.; Whay, H. An investigation into the use of infrared thermography as a rapid diagnostic tool for foot lesions in dairy cattle. *Vet. J.* **2012** 193, 674-678.
67. Church, J.S.; Hegadoren, P.; Paetkau, M.; Miller, C.; Regev-Shoshani, G.; Schaefer, A.; Schwartzkopf-Genswein, K. Influence of environmental factors on infrared eye temperature measurements in cattle. *Res. Vet. Sci.* **2014** 96, 220-226.
68. Barnes, R.J.; Comline, R.S.; Dobson, A. Changes in the blood flow to the digestive organs of sheep induced by feeding. *Q. J. Exp. Physiol*. **1983** Jan;68(1):77-88.
69. Boyd, J.; Ford, E. Normal variation in alanine amino transferase activity in sheep and cattle. *J. Agric. Sci.,* **1967** 68(3), 385-389.
70. Krebs, H. A. Bovine ketosis. *Vet. Rec.* **1966**. 78, 187.
71. Hewagalamulage, S. D.; Clarke, I. J.; Rao, A.; Belinda, A. H. Ewes with divergent cortisol responses to ACTH exhibit functional differences in the hypothalamo-pituitary-adrenal (HPA) Axis, *Endocrinology*, Volume 157, Issue 9, 1 September **2016,** pages 3540–3549.

© 2021 by the authors. Submitted for possible open access publication under the terms and conditions of the Creative Commons Attribution (CC BY) license (http://creativecommons.org/licenses/by/4.0/).
